# Cold Plasma-Treated Ringer’s Saline: A Weapon to Target Osteosarcoma

**DOI:** 10.3390/cancers12010227

**Published:** 2020-01-17

**Authors:** Miguel Mateu-Sanz, Juan Tornín, Bénédicte Brulin, Anna Khlyustova, Maria-Pau Ginebra, Pierre Layrolle, Cristina Canal

**Affiliations:** 1 Biomaterials, Biomechanics and Tissue Engineering Group, Department Materials Science and Metallurgy, Technical University of Catalonia (UPC), Escola d’Enginyeria Barcelona Est (EEBE), c/Eduard Maristany 14, 08019 Barcelona, Spain; miguel.mateu@upc.edu (M.M.-S.); juan.tornin@upc.edu (J.T.); avlada5577@gmail.com (A.K.); maria.pau.ginebra@upc.edu (M.-P.G.); 2 Barcelona Research Center in Multiscale Science and Engineering, UPC, 08019 Barcelona, Spain; 3 Research Centre for Biomedical Engineering (CREB), UPC, 08019 Barcelona, Spain; 4 Inserm, UMR 1238, PHY-OS, Laboratory of Bone Sarcomas and Remodeling of Calcified Tissues, Faculty of Medicine, University of Nantes, 44035 Nantes, France; benedicte.brulin@univ-nantes.fr (B.B.); pierre.layrolle@univ-nantes.fr (P.L.); 5 Institute for Bioengineering of Catalonia (IBEC), Barcelona Institute of Science and Technology (BIST), c/Baldiri i Reixach 10-12, 08028 Barcelona, Spain

**Keywords:** bone cancer, osteosarcoma, cold atmospheric plasma, reactive species, plasma-activated liquid, Ringer’s saline, organotypic model

## Abstract

Osteosarcoma (OS) is the main primary bone cancer, presenting poor prognosis and difficult treatment. An innovative therapy may be found in cold plasmas, which show anti-cancer effects related to the generation of reactive oxygen and nitrogen species in liquids. In vitro models are based on the effects of plasma-treated culture media on cell cultures. However, effects of plasma-activated saline solutions with clinical application have not yet been explored in OS. The aim of this study is to obtain mechanistic insights on the action of plasma-activated Ringer’s saline (PAR) for OS therapy in cell and organotypic cultures. To that aim, cold atmospheric plasma jets were used to obtain PAR, which produced cytotoxic effects in human OS cells (SaOS-2, MG-63, and U2-OS), related to the increasing concentration of reactive oxygen and nitrogen species generated. Proof of selectivity was found in the sustained viability of hBM-MSCs with the same treatments. Organotypic cultures of murine OS confirmed the time-dependent cytotoxicity observed in 2D. Histological analysis showed a decrease in proliferating cells (lower Ki-67 expression). It is shown that the selectivity of PAR is highly dependent on the concentrations of reactive species, being the differential intracellular reactive oxygen species increase and DNA damage between OS cells and hBM-MSCs key mediators for cell apoptosis.

## 1. Introduction

Osteosarcoma (OS) is the most common primary malignant bone tumor and it mainly affects children, adolescents, and young adults. It usually appears as an osteoid-producing solid tumor in the metaphysis of long bones, which experience rapid growth during childhood and adolescence [1]. Despite the low incidence of OS, it is ranked among the most frequent cause of cancer-related child death [2,3]. Current OS therapy consists in surgical resection of the tumor, combined with radiotherapy and/or systemic chemotherapy. Although chemotherapeutic drugs have increased patient survival, this survival rate is still relatively low (50–60%) [4] and many patients develop drug-resistance [5]. On the other hand, chemotherapeutics displays unspecific toxicity with undesired side-effects. For these reasons, innovative therapies are of interest, and thus, cold plasmas could be an interesting approach.

Cold atmospheric plasmas (CAP) are created by generating an electrical discharge and applying it to a gas, and are a source of reactive molecules, ions, electrons, free radicals, UV and electromagnetic fields that have shown a variety of biological effects such as antimicrobial sterilization [6,7], blood coagulation [8] and wound healing [9,10]. CAPs have also been suggested to selectively target cancer cells, showing their efficiency in more than 20 types of cancer without damaging healthy cells and surrounding tissues [11,12].

Plasma-generated electrical fields may induce cell membrane permeabilization and even its disruption [13]. This can act synergistically with the reactive oxygen and nitrogen species (RONS) generated by CAP. There is a wide variety of RONS that can be generated by CAP, which can include short-lived species (ONOO^−^, OH^−^, NO, O_2_^−^, etc.) and long-lived species (O_3_, H_2_O_2_, NO_2_^−^, etc.), and which are thought to be key players in its anticancer effect [14,15,16]. The induction of the increase of intracellular RONS in cancer cells have been reported as a potential target for cancer therapies [17]. Many species are involved in the cellular effects of CAP; for instance, increased levels of hydrogen peroxide are well-known to induce double strand breaks, chromosomal fragmentation and apoptosis in cancer cells [18,19]. In addition, NO_2_^−^ is a precursor for intracellular formation of NO, which induces protein and lipid oxidation, leading to cell death [20].

The selectivity of CAP is suggested to be produced due to the higher sensibility of cancer cells to oxidative stress as a result of their high metabolic rate. This selectivity has been reported to be highly dependent on the composition of RONS generated, which depends on different parameters such as treatment time [21,22], plasma device configuration [23] or gas employed to generate the plasma discharge. The properties of the cancer cells, such as their “activation” state by CAP can also affect their sensitivity to the RONS generated by plasma [24]. For this reason, in this work, two different plasma devices with different design and working gas have been employed to obtain different characteristics of the discharge and of the treated liquid.

In an in vitro situation, RONS produced in the plasma are transported to the liquid covering the cells and react with this liquid forming secondary reactive species [25]. In this sense, the effects of plasma can be triggered by direct application over cells or by previous treatment of the liquid media as an indirect treatment [26,27]. This second approach is more interesting for OS, as it would avoid the open surgery needed to expose the tumor to CAP. Taking into account that in clinics drugs are supplied dissolved in saline solutions, the possibility of using this kind of solutions as plasma-activated liquids is an attractive therapeutic option that has been explored with interest for glioblastoma, cervical and ovarian [11] and pancreatic [28] cancers (among others) but is still unexplored for OS, and is undertaken in this work.

In recent years, CAP have shown lethal in vitro efficiency by direct treatment as well as by plasma-treated cell culture medium in different OS cell lines and by different plasma devices [22,23,29,30,31]. OS is a heterogeneous disease characterized by presenting different subtypes and high levels of genomic instability [32,33], making it difficult to assess the clinical relevance of new therapeutic options for the different cases. For example, two main tumor suppressor proteins (P53 and pRB,) are suggested to be altered in over 50% of patients [34], and are involved in cell cycle arrest, DNA repair and induction of apoptosis [35]. Given the heterogeneity of OS, it is of paramount interest to evaluate OS cell lines with different genomic profiles in views of assessing the real applicability of plasma-activated liquids in OS. Our previous work employing SaOS-2 cells (which are p53 and pRB null), highlighted the importance of an equilibrated cocktail of RONS for anticancer selectivity of the plasma-activated medium and revealed the critical role of H_2_O_2_ in these cytotoxic effects [36].

From another point of view, up to now, cytotoxicity of CAPs or plasma-activated media in OS cell lines have been investigated only in adherent cell cultures [22,23,29,30,31,36,37]. This kind of in vitro models provides limited clinically relevant information. In 2D models, some key aspects like the impact of stroma and cell-to-cell interaction are lacking [38,39,40]. The effects of CAP have been reported to be highly dependent of the number of cells and 3D spatial distribution [41,42]. In order to evaluate the applicability of CAP-based therapies, it is necessary to move from 2D cell cultures to 3D models to have a more relevant situation.

In light of the facts discussed above, the objective of this work is to investigate for the first time the cytotoxic effects of plasma-activated Ringer’s saline (PAR) in OS cell cultures and its possible translation to a 3D OS model while providing insights on the mechanism potentially involved. To that aim, firstly, the cytotoxic potential of PAR obtained from two different atmospheric pressure plasma jets is evaluated in human OS cell lines with different genetic profile; SaOS-2 (p53−/pRB−), U2-OS (p53+/pRB−) and MG-63 (p53−/pRB+) [43], and in human Bone Marrow Mesenchymal Stem Cells (hBM-MSCs). This is investigated with regard to the RONS generated in PAR by plasma jets and their intracellular effects. Secondly, a tumor was generated in mice by paratibial injection of MOS-J cells and the effects of PAR are investigated in sections of the tumor through organotypic cultures.

## 2. Results

### 2.1. Characterization of the Plasma Gas Phase and Effects Producing PAR

Two different plasma devices were employed in this work to treat Ringer’s saline and obtain PAR; a single-electrode needle device operating in helium that will herein be designated as atmospheric pressure plasma jet (APPJ) and the kINPen, which is a pin-type electrode with a grounded outer electrode operating with Argon.

**Figure 1:**
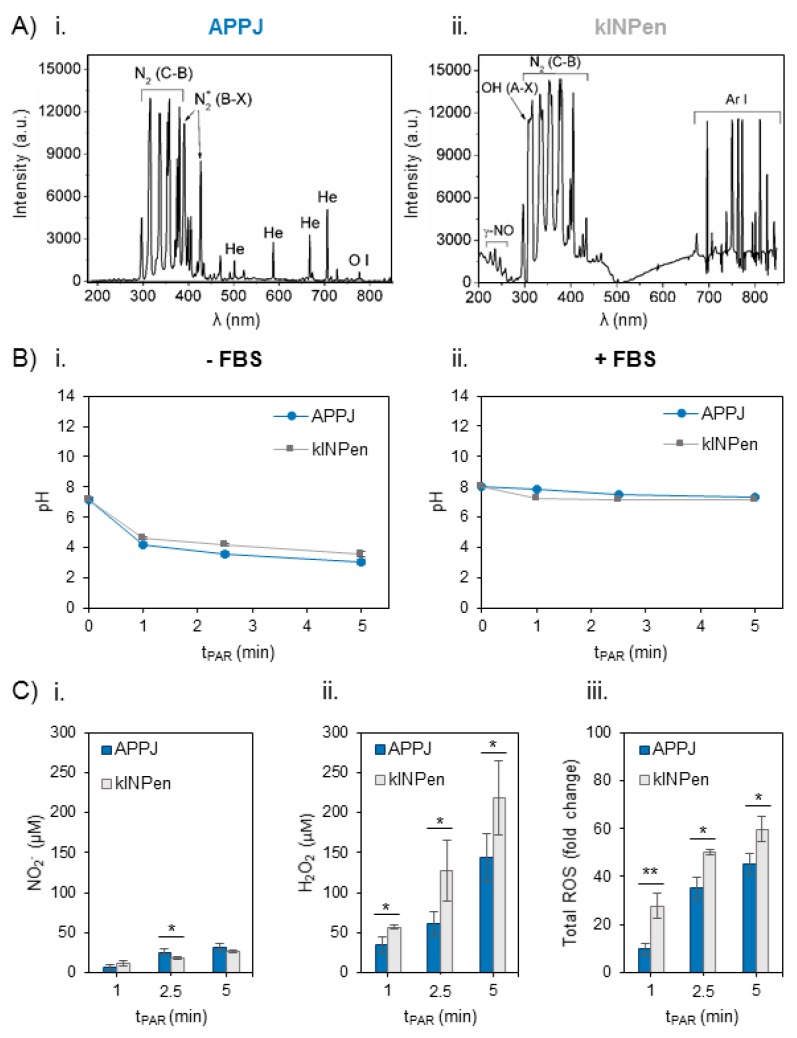
Reactive oxygen and nitrogen species in the plasma gas phase and time-dependent concentration generated in Ringer’s saline at 1 L/min of gas flow, 10 mm of distance between the nozzle of the plasma jet and the surface of the liquid (2 mL in 24 well-plates). (**A**) Optical Emission Spectra (OES) of the plasma gas phase in (**Ai**) atmospheric pressure plasma jet (APPJ) and (**Aii**) kINPen during treatment of Ringer’s saline. (**B**) pH evolution in Ringer’s saline treated by APPJ and kINPen at increasing treatment time (**Bi**) without and (**Bii**) with addition of 10% FBS after treatment. (**C**) Concentration of (**Ci**) NO_2_^−^, (**Cii**) H_2_O_2_ and (**Ciii**) total reactive oxygen species (ROS) measured in situ and relativized to untreated plasma-activated Ringer’s saline (PAR). Reactive oxygen and nitrogen species (RONS) created by both plasmas in 2 mL of Ringer’s saline were measured right after plasma treatment and addition of 10% of FBS. Asterisks represent statistically significance differences between APPJ and kINPen for each time-point (*n* = 3; * *p*-value < 0.05; ** *p*-value < 0.01; two-sided Student’s *t*-test).

Optical Emission Spectra were obtained for both plasma jets employing the same parameters of gas flow (1 L/min) and distance between the nozzle of the jet and the surface of the liquid (10 mm) during treatment of the Ringer’s saline (Figure 1A). Apart from the main differences regarding the Helium or Argon emission bands in APPJ or kINPen respectively, both jets show emission bands from oxygen and nitrogen species. The most intense bands in APPJ come from Nitrogen, namely N_2_ 2nd positive system, N_2_^+^ 1st negative, are recorded, as well as monoatomic oxygen (at 777 nm) (Figure 1(Ai)). kINPen shares only N_2_ 2nd positive in its emission bands, while other relevant species appear: OH and γ-NO radicals (Figure 1(Aii)).

Secondly, the evolution of pH with plasma treatment time was measured in Ringer’s saline, which progressively decreased from pH 7.4 to 3 with both plasma jets (Figure 1(Bi)). Addition of 10% FBS right after plasma treatment (necessary for cell culture studies) buffered the plasma-induced decrease, so pH after 5 min of plasma treatment was recorded to be 7.15 (Figure 1(Bii)).

The concentration of NO_2_^−^ and H_2_O_2_ and total reactive oxygen species (ROS) in situ production were measured in Ringer’s saline treated by APPJ and kINPen from 1 to 5 min just after the addition of 10% FBS (Figure 1C). The generation of H_2_O_2_ and NO_2_^−^ and their concentration increase were treatment time-dependent in PAR with both devices. Few differences were recorded among APPJ or kINPen in the concentration of NO_2_^−^, which was much lower than that of H_2_O_2_ (Figure 1(Ci)). The amount of H_2_O_2_ generated was significantly higher (*p* < 0.05) with kINPen than APPJ, ranging from 40 µM to 150 µM for APPJ and from 60 µM to 220 µM for kINPen (Figure 1(Cii)). Total reactive oxygen species (ROS) were measured in plasma-treated Ringer’s saline in situ, following the same trend observed for peroxides, with significantly higher amounts formed with kINPen than with APPJ up to 5 min (*p* < 0.01 for 1 min and *p* < 0.05 for 2.5 and 5 min) (Figure 1(Ciii)).

### 2.2. Effects of PAR on 2D Cultures of Human OS Cell Lines and hBM-MSCs

**Figure 2:**
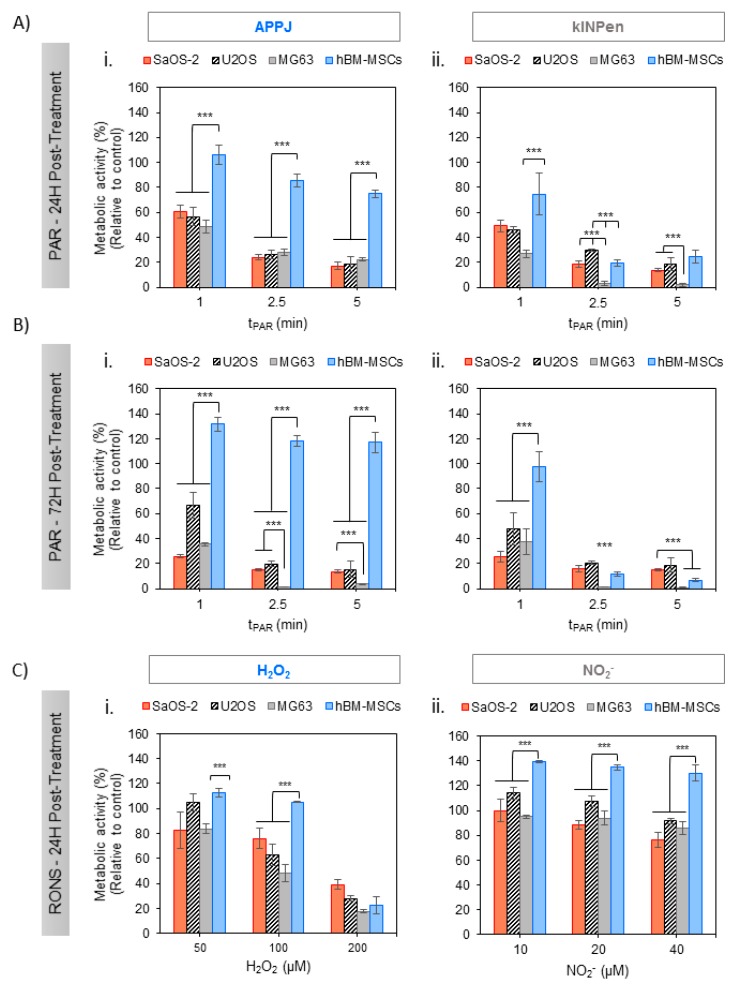
Effects of PAR on the metabolic activity of human OS cell lines (SaOS-2, MG-63, U2-OS) and healthy hBM-MSC with treatment time. Cells in adherent culture were exposed during 2 h to PAR treated by APPJ or kINPen for 1, 2.5 and 5 min. After that, PAR was replaced by fresh medium. Metabolic activity was determined 24 h (**A**) and 72 h (**B**) after PAR exposure by WST-1 test. (**C**) Cells were also exposed during 2 h to increasing concentrations of H_2_O_2_ and NO_2_^−^ standards in Ringer’s saline with 10% FBS (which match with concentrations determined in Figure 1), corresponding to 50, 100 and 200 µM for H_2_O_2_ (**Ci**) and 10, 20 and 40 µM for NO_2_^−^ (**Cii**) and metabolic activity was determined 24 h after exposure. Values were relativized to cells exposed to untreated PAR. Asterisks represent statistically significant differences among cell lines for the same PAR treatment time-point. (*n* = 3; *** *p*-value < 0.005, ANOVA and two-sided Student’s *t*-test).

PAR was obtained from Ringer’s saline treated with APPJ and kINPen at increasing treatment times and, after addition of 10% FBS, it was placed in contact with adherent OS cells for 2 h. Metabolic activity of SaOS-2, U2-OS, MG6-3 and healthy hBM-MSCs (Figure 2) revealed interesting effects: On the one hand, both plasma jets efficiently reduced metabolic activity in all human OS cell lines 24 h after exposition in a plasma treatment time-dependent manner (Figure 2A). The effects of this single treatment with PAR were fostered at 72 h, being more evident in MG-63 cells which attained complete cell death already with a 2.5 min treatment (Figure 2B). The cytotoxicity induced by PAR obtained from either two of the plasma jets employed followed essentially the same trends. Nevertheless, kINPen seemed to produce more cytotoxic PAR than APPJ, as it yielded slightly lower metabolic activity, especially at 24 h, with MG-63 cells already dead in 2.5 and 5 min treatments (Figure 2(Aii)).

On the other hand, healthy hBM-MSCs display completely distinct behavior following contact with APPJ-treated PAR (Figure 2(Ai,Bi)); they show significantly higher cell viability than OS cell lines, between 100 and 80% up to 5 min of treatment at 24 h (*p* < 0.005 for all treatment times) and recovering their complete viability and even proliferating between 120 and 130% after 72 h (*p* < 0.005). Conversely, kINPen–treated PAR induced deleterious effects in hBM-MSCs treated with PAR from 2.5 min of plasma treatment at all incubation times, while hBM-MSCs treated with 1 min PAR kept their complete viability. Given the interest in developing selective treatments, subsequent experiments focused on APPJ-treated PAR as it ensured healthy cell survival while being lethal for OS.

**Figure 3:**
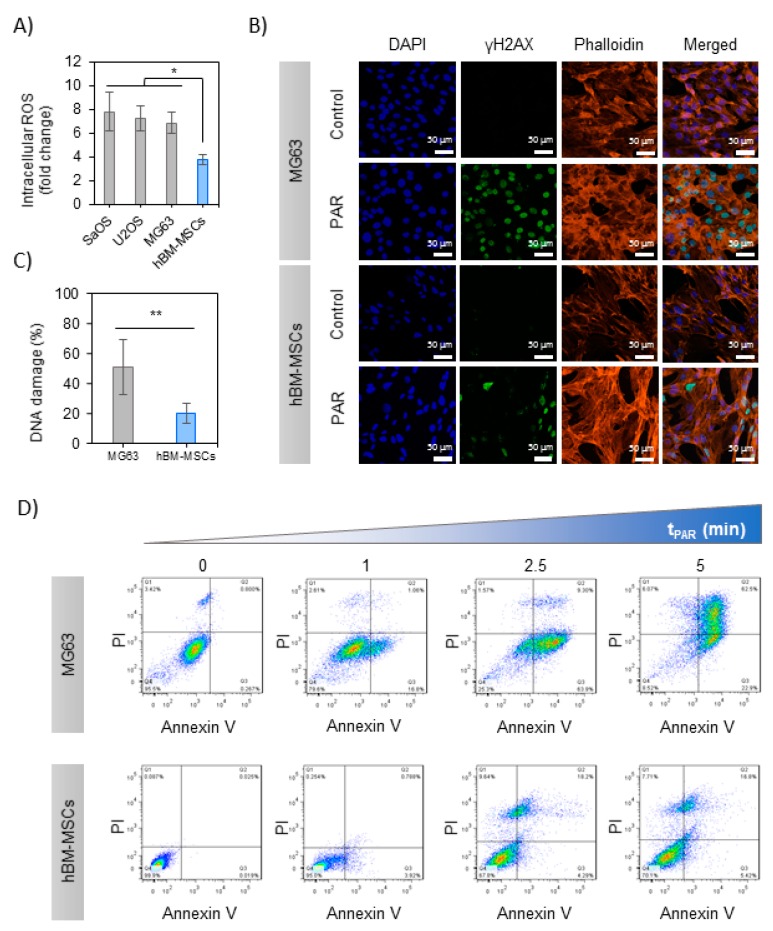
PAR-induced selective cell death in MG-63 cells rather than in hBM-MSCs. (**A**) Intracellular ROS measurement; OS cell lines and hBM-MSCs were incubated with dichlorofluorescin diacetate (DCFH-DA) and exposed during 2 h to PAR treated during 5 min by APPJ and after that fluorescence was measured. Values were relativized to negative control. (**B**) % of DNA damage quantification (positive γH2AX area relativized to DAPI area; images: *n* = 5, mean of 70 nuclei per image). Asterisks represent statistically significance (*n* = 3; * *p*-value < 0.05; ** *p*-value < 0.01; ANOVA and two-sided Student’s *t*-test). (**C**) Representative images of MG-63 and hBM-MSCs cells after 2 h of exposition to 5 min-treated PAR by APPJ. Cells were labelled with DAPI (nuclei, blue), phalloidin (F-actin, orange) and anti-γH2AX (DNA damage reporter, green). Scale bar = 50 µM. (**D**) MG-63 and hBM-MSCs cultures were exposed during 2 h to untreated PAR and treated during 1, 2.5 and 5 min with APPJ. After that, PAR was replaced by fresh medium. Cells were collected 24 h after PAR exposure and then they were stained with Annexin-V/PI and analyzed by flow cytometry. This assay was done in triplicate.

The levels of intracellular ROS were measured in the cells exposed to 5 min-treated PAR with APPJ, revealing differential effects between OS and hBM-MSCs 2 h after exposure to PAR (Figure 3A): while PAR induced an increase in intracellular ROS in both OS cells and healthy cells, in OS the rise was 7–8 times higher than the control. In contrast, a much more moderate increase was recorded in hBM-MSCs 4 times higher than control, finding significant differences between OS and hBM-MSCs (*p* < 0.05). Given the increased cytotoxic effects in MG-63 and similar intracellular ROS generated by exposition to PAR, this cell line was selected for subsequent analysis of the possible mechanisms involved.

In the first place, DNA damage was evaluated by immunostaining of γH2AX foci in MG-63 and hBM-MSCs 6 h after the treatment with PAR (Figure 3B). As reflected in the image, only PAR-treated cells display green immunostaining indicating DNA damage in the nuclei; this was quantified by the increase of γH2AX/DAPI ratio being significantly higher in MG-63 cells (51 ± 18%) than in hBM-MSCs (20 ± 7%) (*p* < 0.001) (Figure 3C).

Secondly, the mechanism of cell death was evaluated by flow cytometry in MG-63 and hBM-MSCs cells exposed to increasing treatment times of PAR. PAR treatment decreased the percentage of alive (Anx−/PI−) cells in a dose-dependent manner (Figure 3D), leaving less than 10% of alive cells in 5 min treated PAR. Treatment time of PAR up to 2.5 min progressively led to pre-apoptotic cells (Anx+; *p* < 0.005). The percentage of late-apoptotic cells (Anx+/PI+) was above 70% with 5 min-treated PAR (*p* < 0.005), and no necrotic cells (Anx−/PI+) were recorded in any case (Supplementary Figure S1A). In contrast, hBM-MSCs just showed a minor decrease in cell viability (70% of Anx−/PI− up to 5 min; *p* < 0.005), still displaying essentially apoptotic cell death mechanisms (Figure S2B).

### 2.3. Effects of PAR in a 3D Model: Murine OS Tumor Sections

**Figure 4:**
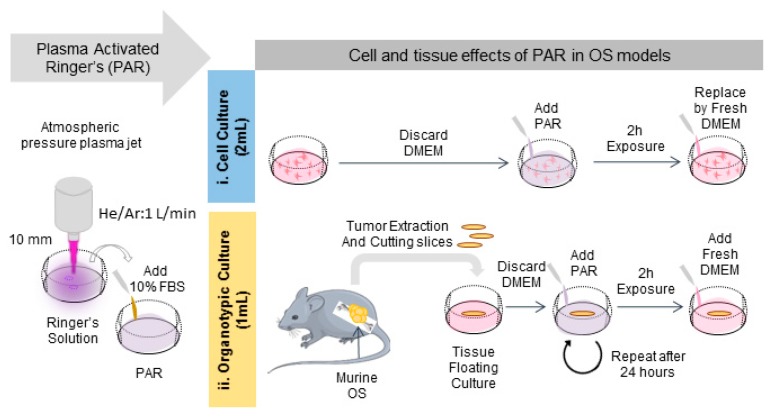
Protocol followed for the generation of PAR and the treatment of cell and organotypic cultures. Briefly, PAR was obtained by treatment of Ringer’s saline at different times followed by adding 10% FBS. For cell cultures (**i**), cells were placed with 2 mL-treated PAR and incubated during 2 h; after that PAR was replaced by fresh medium. For organotypic cultures (**ii**), murine OS tumors were cut in slices and placed in floating culture; then samples were placed with 1 mL-treated PAR during 2 h and after that, PAR was diluted at 25% in fresh medium. In this case, treatment was repeated after 24 h.

**Figure 5:**
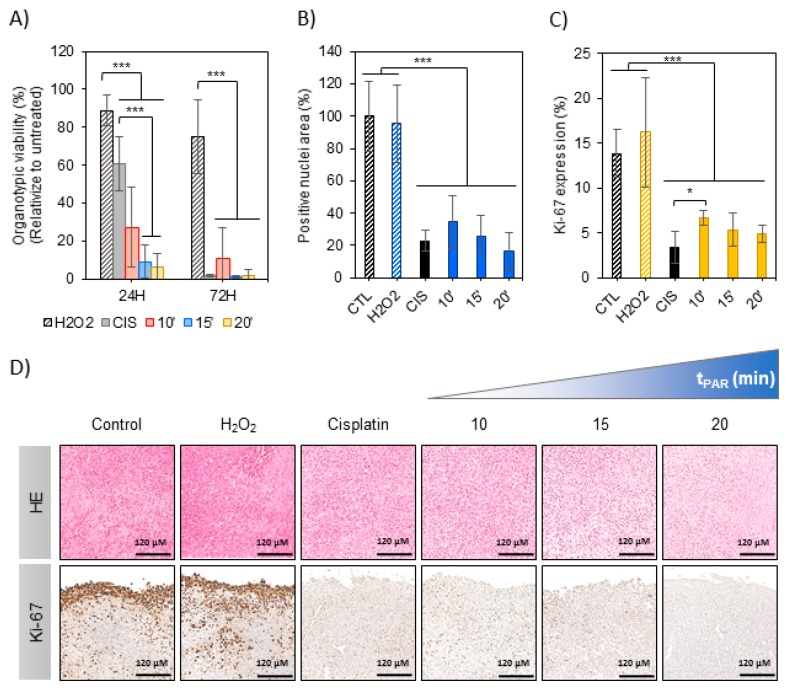
PAR effects in mouse organotypic OS model. Mouse OS tumor sections in floating culture were treated with PAR at increasing treatment times (10, 15 and 20 min) or by 100 µM of Cisplatin (CIS) or 500 µM of H_2_O_2_. (**A**) Metabolic activity was determined by resazurin 24 and 72 h after PAR exposure. In this assay, four sections were analyzed per condition, and the assay was performed in three independent experiments. Values were relativized to tumor sections exposed to untreated PAR. Tumor sections were fixed 72 h after treatment and processed for histological analysis. Samples were stained with haematoxylin/eosin (HE) and immunostained for Ki-67. (**B**) HE quantification of positive nuclei stained areas (values relativized to control). (**C**) Percentage of positive Ki-67 stained nuclei. For statistical analysis, 5 well-distributed images were taken at X40 and were analyzed. Asterisks represent statistically significance between different conditions (*n* = 3; * *p*-value < 0.05; *** *p*-value < 0.005; ANOVA and two-sided Student’s *t*-test). (**D**) Representative images for HE and Ki-67 immunostaining for each condition (Scale bar = 120 µm).

In this section the effects of PAR were evaluated on organotypic cultures to count with a 3D model of bone cancer. An OS tumor generated in intramuscular paratibial site of mice was excised and after obtaining regular tumor sections they were exposed to PAR (Figure 4). A significant reduction of metabolic activity was observed by exposition of the tumor sections to PAR as a function of plasma treatment time, beyond the values obtained with cisplatin 24 h after treatment (*p* < 0.005) (Figure 5A), especially for PAR treated during 15 and 20 min. Metabolic activity was completely abrogated 72 h after the treatment, both with PAR or with Cisplatin. No significant effects were observed by 500 µM H_2_O_2_, which was an equivalent concentration to PAR treated for 15 min (Figure S2B).

Haematoxylin/Eosin (HE) and Ki-67 immunostaining allowed to evaluate histological effects of PAR in tumor tissues after 72 h of treatment (Figure 5D). A significant reduction of relative nuclei area (HE staining) was observed to be induced by PAR with increasing plasma treatment times (*p* < 0.005) (Figure 5B). This reduction was also observed in Cisplatin-treated samples, being of more than 70% (Figure 5B). A high number of proliferating cells (Ki-67 stained) were observed in the first 40 µm of tumor section margins in the control (Figure 5D), with less percentage of Ki-67 cells in the rest of the section (14 ± 3%) (Figure 5C). Proliferation in tumor margins was abrogated by both PAR and Cisplatin at 72 h (Figure 5D); the percentage of Ki-67 expressing cells was significantly reduced by PAR treatment, being of 5–7% for PAR-exposed sections (Figure 5C; *p* < 0.005). The reduction of proliferating cells by Cisplatin was equivalent to 15–20 min of PAR. In contrast with the results in 2D, no effects were observed to be induced by H_2_O_2_ added at the 500 µM of concentration neither in proliferation nor in nuclei area.

## 3. Discussion

We report here on the effects of saline solutions activated by plasma in 2D adherent and on 3D organotypic OS cultures, employing a clinically compatible Ringer’s saline. Cold atmospheric plasmas have been highlighted as a potential therapy against many types of cancers. However, most studies up to now were conducted in vitro (95%), mostly in 2D cultures, and only 26% evaluated the effects of plasma-activated liquids on the cells (as opposed to directly treating the cells with plasma) [44]. The direct application of plasma and the homogeneity of the treatment may depend on tumor size and body location, restricting the possibility of treating tumors hard to reach. Employing plasma-activated liquids can allow injection to the tumor site, and thus provide a minimally invasive approach for this therapy [11].

The reactivity transfer from the plasma gas phase to the liquid phase has been highlighted as being of prime importance for its biological effects [21]. Despite employing here two jets that use noble gases (Helium in APPJ or Ar in kINPen), an important part of the reactivity of these jets derives from mixing of the plasma gas phase with air. The gas phase of the APPJ during treatment of Ringer’s included different nitrogen species, monoatomic oxygen, and hydroxyl radicals, as recorded by optical emission spectroscopy (Figure 1(Ai)), as similarly observed during treatment of cell culture medium [22,36]. Both plasma jets have been extensively characterized in [45,46]. In contrast, kINPen produced two other oxygen-containing species: γ-NO and OH (Figure 1(Aii)). These species in the gas phase were related to a progressive increase of RONS in Ringer’s with plasma treatment time. In fact, while similar amounts of NO_2_^−^ were generated with both jets, much higher concentrations of H_2_O_2_ and of total ROS were recorded in PAR produced from kINPen (Figure 1C).

In the presence of air, short-lived reactive nitrogen species such as (NO or ONOO^−^) are formed in liquid medium. These nitrogen oxides subsequently react in water forming acids, which affect the conductivity and pH of plasma-treated liquids and based on pH dissociate to nitrite (NO_2_^−^) (Figure 1C) and nitrate (NO_3_^−^) ions. In fact, decrease of pH (from 7.3) to non-physiological values (3–4) was recorded after treatment, in a dose-dependent manner (Figure 1(Bi)), as observed in other works employing water or non-buffered saline solutions [47,48]. An important body of literature led by Hori et al. have proposed clinical-compatible saline solutions to be activated by cold plasmas, such as Ringer lactate solutions [11]. These solutions present some experimental limitations for in vitro conditions, mainly the lack of nutrients to sustain cell viability [15,48]. For this reason, here 10% of FBS was added to Ringer’s to mimic nutrient tumor environment and allow further treatment of cells with this liquid. To avoid altering the reactivity of FBS with plasma [41], it was added just after plasma treatment. The addition of 10% of FBS after treatment stabilized the pH between 7.8 and 7.1 (Figure 1(Bii)), which allowed isolating the cell effects due only to the concentration and kind of RONS generated in the liquid, as in non-buffered solutions high pH decrease induced by plasma treatment is also reported to severely compromise cell viability [12,48].

It was shown in previous works [36,49] that long treatment time of cell culture medium may produce a lethal concentration of H_2_O_2_ which overwhelms cell defense mechanisms and kills both OS and healthy cells [36]. In line with these data, to obtain an equilibrated cocktail of RONS, plasma treatments only up to 5 min were selected (Figure 1C). Due to the low stability of NO_2_^−^ produced by plasma (Figure S1), cell cultures were exposed to PAR just after production. In our experiments, incubation of cell cultures or tumor sections in contact with PAR for only 2 h was enough to induce cytotoxic effects in SaOS-2, MG-63 and U2-OS cells (Figure 2). The different p53 and pRB status of these human OS cell lines (p53−/pRB− for SaOS-2, p53+/pRB− for U2-OS and p53−/pRB+ for MG-63) [44] indicates that plasma-activated Ringer’s cytotoxic action possibly follows a p53 and pRB− independent mechanism of cell death.

Cold atmospheric plasmas are reported to act selectively targeting cancer cell lines without affecting their healthy homologues [50,51]. This can be explained by the gap between cancer cells and normal cells, due to an increased cell metabolism and intracellular production of RONS [52] which oversaturates antioxidant system, making cancer cells more sensitive to oxidative stress [53]. The ability of PAR targeting different OS cell lines but without deleterious effects in hBM-MSCs is clearly observed in this study, in Ringer’s obtained from treatment with APPJ but not with kINPen (Figure 2). At discussed earlier, under these working conditions kINPen produced higher amount of total ROS and H_2_O_2_ (i.e., 219 ± 46 μM at 5 min) than APPJ (143 ± 30 μM at 5 min), but without significant differences in the production of NO_2_^−^. This higher concentration of H_2_O_2_ produced in PAR by kINPen in contrast with APPJ may compromise PAR selectivity. However, the fact that the concentration of H_2_O_2_ produced by kINPen at 2.5 min treatment is equivalent to that of 5 min of APPJ treatment but the selectivity of the treatment is lost only for kINPen indicates that other species apart from those measured here are formed in Ringer’s that are important for cell survival. As mentioned earlier, it has been described that a wide variety of the species generated by CAP can be of influence in its anticancer effects (i.e., ONOO^−^, OH^−^, NO, O_2_^−^, O_3_, H_2_O_2_, NO_2_^−^, NO_3_^−^, etc.) [14,15,16].

Controls were prepared with artificially added H_2_O_2_ or NO_2_^-^ at concentrations equivalent to those of kINPen and showed that NO_2_^−^ does not affect cell toxicity in OS cell lines and increase cell viability in healthy cells (Figure 2C). Equivalent concentrations of H_2_O_2_ induced less cytotoxic effects than PAR in OS, suggesting again that other RONS are contributing to the cytotoxic effects produced by PAR. Recent studies have shown that artificially added H_2_O_2_ and NO_2_^−^, which lead to the formation of peroxinitrite, act synergistically and produce higher cytotoxic effects than each species separately [54].

The highest H_2_O_2_ concentration leads, as in kINPen-treated PAR, to a loss of selectivity (hBM-MSC cell death). To obtain an equilibrated cocktail of RONS and avoiding a loss of anti-cancer selectivity [36], for further work we selected APPJ-treated PAR. In order to adjust the production of RONS, plasma parameters like gas flow [36,55], distance of the nozzle [7,36,55], working gas composition [56,57,58,59] and here, device configuration [60,61] are of paramount importance to adjust the composition of RONS generated in the liquid, and the conditions selected here followed previous works of our group [36,62].

The genotoxic potential of PAR was evaluated by analyzing the levels of intracellular ROS and γH2AX and apoptosis activation. The three OS lines analyzed display similar 7-fold increase in intracellular ROS, while the rise recorded in healthy cells is only about half of that from OS cells (Figure 3A), which can be explained by the decreased antioxidant ability of cancer cells [17]. APPJ-treated PAR induced an increase in the level of γH2AX in both healthy and OS cells after treatment (Figure 3B,C), in line with previous works [36,56,63]. The lower levels of γH2AX recorded after PAR treatment in hBM-MSCs indicate that PAR induces DNA damage mainly in human OS cells. Following the mentioned results, there seems to be a clear correlation between the increase in the intracellular ROS and the induction of DNA damage. A relevant fact is APPJ-treated PAR induces apoptosis in MG-63 cells (Figure 3D), as observed in previous works for other OS cells treated with plasma-activated cell culture media or even directly with plasma [22,36]. On the other hand, hBM-MSCs are clearly less affected, with little induction of late apoptosis and necrosis at longer APPJ treatment times on PAR (this is quantified in Figure S2).

In recent years, 3D cultures are gaining increasing interest due to the need to recapitulate the complexity of the in vivo situation. As reported in previous studies, tumor complexity regarding the presence of stroma, cell-to-cell contact [64], and tumor heterogeneity [65,66] have a high impact in chemotherapy efficiency [67,68] and also on the penetration of RONS [69]. To take all this into account, the treatments on tumors were performed by two doses of PAR separated by 24 h, and to obtain higher concentration of RONS in plasma-activated media, plasma treatment was performed in lower volume of saline solution and longer treatment times (Figure S3). The treatment profile designed showed effectiveness to completely reduce metabolic activity of tumor sections up to 72 h (Figure 5A).

We confirmed the anti-tumoral potential of PAR by Hematoxylin-Eosin (HE) and Ki-67 immunodetection, showing that PAR can induce cell death and decrease of proliferation in a similar way than 100 µm Cisplatin (Figure 5A–C). In 2D we observed that concentrations of H_2_O_2_ equivalent to those generated with plasma treatment had clear cytotoxic effects (Figure 2C), so 500 µM H_2_O_2_ was employed as a positive control (Figure 5B–D). In the 3D organotypic cultures it was clear that H_2_O_2_ did not induce any cell death, nor decrease on Ki-67 (Figure 5A–C). It was also observed that caspase-3 (caspase protein involved in mitochondrial apoptotic pathway) was induced by Cisplatin but not by PAR (Figure S4), suggesting a caspase-3 independent mechanism (which does not exclude apoptosis, but rather indicates that other markers are involved in the process). Our results reflect on the important effect of PAR as potential anti-tumoral therapy, which reduces the viability of the ex vivo tumor sections by reducing cell proliferation in a similar way to high doses of Cisplatin on OS models with the advantage of not affecting healthy hMSCs.

## 4. Materials and Methods.

### 4.1. Cell Lines

Sarcoma osteogenic SaOS-2, MG-63 and U2-OS cells (ATCC, Manassas, VA, USA) were cultured in DMEM (Gibco^TM^, Carlsbad, CA, USA) supplemented with 10% of fetal bovine serum (FBS), 2 mM L-glutamine and penicillin/streptomycin (50 U/mL and 50 µg/mL, respectively), all from Gibco^TM^. Cells from passages 1–29 were used in all experiments. hBM-MSCs (ATCC, USA) were expanded in Advanced DMEM (Gibco^TM^) supplemented with 10% of FBS, 2 mM L-glutamine and penicillin/streptomycin. Cells from passages 1–9 were used in all experiments. MOS-J cell line, established from spontaneous C57BL/6J mouse OS by Joliat, M.J. et al. [70], were expanded in DMEM with glucose at 4.5 g/L (Lonza, Walkersville, MA, USA), supplemented with 1% of FBS, and 1% penicillin/streptomycin. For organotypic culture, DMEM with 15% FBS and 1% penicillin/streptomycin was prepared and glucose was added to the medium to each a final concentration of 6 g/L. The same medium was used for 2D and 3D plasma treatments. All cell lines were maintained and expanded at 37 °C in a 95% humidified atmosphere containing 5% of CO_2._

### 4.2. Atmospheric Pressure Plasma Jets

Two atmospheric pressure plasma jets were employed: An atmospheric pressure plasma jet (APPJ) was created using He (5.0 Linde, Spain) as plasma carrier gas in a jet design with a single electrode as described elsewhere [46]. The discharge electrode was a copper wire with a diameter of 0.1 mm inserted inside of a 1.2 mm inner diameter quartz capillary tube, covered by a polytetrafluoroethylene (PTFE) holder. The electrode was connected to a commercial high voltage power supply from Conrad Electronics (nominally 6 W power consumption). The discharge was operating with sinusoidal waveform at 25 kHz with (U) ~2 kV and (I) ~3 mA. Helium flow in the capillary was regulated by a Bronkhorst MassView flow controller [22].

kINPen^®®^ IND (Neoplas tools GmbH, Greifswald, Germany) is a commercial plasma jet tool that consists of a hand-held unit that discharges plasma under atmospheric conditions, employing a DC power unit and Argon gas to generate the plasma. In the centre of a ceramic capillary (inner diameter 1.6 mm) a pin-type electrode (1 mm diameter) is mounted, and a ring around the dielectric as grounded counter-electrode. The needle is powered by a small RF generator producing a sinusoidal voltage waveform ranging from 2 kV to 3 kV amplitude peak at a frequency of 1 MHz and modulated with 2.5 kHz and a plasma duty cycle of 1:1 [71,72].

Both plasma jets operated with a gas flow of 1 L/min, based on a previous work [36] at a distance of 10 mm between the surface of the liquid and the jet nozzle.

### 4.3. Optical Emission Spectroscopy (OES)

OES was used to determine the main plasma emitting species. The equipment used was a spectrometer F600-UVVIS-SR (StellarNet, Tampa, FL, USA), which was connected to an optical fiber (QP600-2SR-Ocean Optics) with lens that collected information from the measure point near the plasma jet (integration time 10,000 ms, average of 5 scans). Measurements were conducted close to the surface. For data processing, the SpectraWiz software (StellarNet, Tampa, FL, USA) was used.

### 4.4. Plasma Treatment

To produce the plasma-activated Ringer’s saline (PAR), 2 mL of sterile Ringer’s saline (8.6 g/L NaCl, 0.33 g/L CaCl_2_ and 0.3 g/L KCl) were placed under the jet at room temperature at a distance of 10 mm from the jet nozzle in sterile conditions. The liquid was placed in multiple well plates of 1.9 cm^2^ of surface (24 well-plates). Plasma treatment times between 1–5 min were investigated. A 10% of FBS was added immediately after treatment. The protocol followed is schematized in Figure 4. For treatment of organotypic cultures, 1 mL of Ringer’s saline were treated with APPJ between 10–20 min at same conditions.

### 4.5. Characterization of PAR

For characterization of PAR, each parameter was measured immediately after plasma treatment and addition of FBS. pH was measured using an MM 41 Crison multimeter. The H_2_O_2_ produced by plasma was measured using an Amplex Red/horseradish peroxidase method (all from Sigma) and by monitoring the peak of fluorescence at λex/em of 560/590 nm. H_2_O_2_ concentrations were determined using a calibration curve constructed from known stock H_2_O_2_ solutions (0–10 μΜ). NO_2_^−^ were quantified by using the Griess reagent method and using a calibration curve from known NaNO_2_ stock solutions (0–100 μΜ). Griess reagent is prepared from sulphanilamide (1%), *N*-(1-naphtyl) ethylene diamine (NEED) (0.1%) (both from Sigma), phosphoric acid (1.2%) (Panreac) and distilled water (97.7%). The absorbance was measured at 540 nm. For total ROS detection, 2′,7′-dichlorofluorescin diacetate (DCFH-DA; Sigma) was used. Briefly, 150 μL of sample were placed in black 96 well-plates and 1 μL of DCFH-DA at 2 mM in DMSO was added to each sample. Samples were incubated for 30 min at 37 °C and then fluorescence intensity was read at a λex/em of 485/528 nm. Total ROS increase was expressed as a fold change. In total ROS detection all the reactive oxygen species present in the media are considered, therefore including short and long-lived species (i.e., H_2_O_2_, NO_2_^−^, HOO *, * OH, NO *, ONOO *, etc.). Absorbance and fluorescence were measured using a Synergy HTX multi-mode microplate reader (BioTek Instruments, Winooski, VT, USA). From all techniques, samples were measured in triplicate.

### 4.6. Metabolic Activity

Subconfluent SaOS-2, MG-63, U2-OS and hBM-MSCs were trypsinized, centrifuged and seeded in a 48-well plate at a density of 15x 10^3^ cells/well, and incubated in 300 μL of their corresponding medium for 24 h. After incubation, the culture medium was replaced in each cell line by 300 µL of PAR treated during 1, 2.5 or 5 min where 10% FBS had been added immediately after treatment. Cells were incubated during two hours with PAR for each condition in triplicate. As a positive control, each cell line was incubated during two hours with untreated PAR. Afterwards, PAR was replaced by fresh complete DMEM. The experimental protocol is shown in Figure 5. Cells were then incubated at 37 °C for 24 h and 72 h. Cell metabolism was evaluated by WST-1 assay (Roche, Mannheim, Germany) 18 µL of WST-1 were employed per mL of culture medium. As a negative control, WST-1 was incubated without cells. The absorbance was measured at 440 nm and absorbance from negative control was subtracted. Absorbance of each treated condition was referenced to positive control.

### 4.7. Immunofluorescence

For immunofluorescence staining, MG63 and hBM-MSCs cells were incubated during 2 h with PAR treated during 5 min. After incubation, PAR was removed and changed by fresh medium, cells were incubated during 6 h and then fixed with 4% of paraformaldehyde (Sigma) for 20 min at room temperature. Then, cells were washed, twice with PBS, permeabilized with 0.1% Triton X-100 in PBS and blocked with 5% BSA in PBS-0.1% Tween-20 10X (blocking solution) with agitation during 3 h. Then, cells were washed and incubated with mouse Anti-phospho-Histone γH2AX (Ser139) Antibody, clone JBW301 (Merk Millipore, Burlington, MS, USA) (1:500 in blocking solution) in agitation at 4 °C overnight and after washing with PBS, cells were incubated with secondary antibody Alexa Fluor 488 goat anti-mouse (Invitrogen) for 30 min in the dark. After that cells were washed with PBS and incubated with Alexa Fluor 546 Phalloidin (1:300 in PBS-0.1% Tween-20) (Invitrogen Carlsbad, CA, USA) during 1 h in dark. Cells were washed with 20 mM glycine in PBS and samples were mounted with ProLong™ Gold Antifade Mountant with DAPI (LifeTechnologies, Carlsbad, CA, USA). Images were captured using Zeiss laser scanning microscope. For % of DNA damage, total positive area for γH2AX foci and DAPI stain were quantified using ImageJ software and total γH2AX-positive area were relativized to total DAPI area (*n* = 5, with a mean of 70 nuclei per image).

### 4.8. Intracellular ROS Measurement

Intracellular levels of ROS were measured using DCFH-DA. SaOS-2, MG63, U2OS and hBM-MSCs cells were cultured in black 96-well plates at a density of 5000 cells/well in their corresponding medium for 24 h. After that, cells were incubated before treatment during 1 h with 40 µM of DCFH-DA in DPBS, prepared from 2 mM of DCFH-DA solved in DMSO. Afterwards, cells were washed in DPBS and treated with PAR for 2 h following the protocol in Figure 4i. To quantify of intracellular ROS levels, fluorescence was measured after 2 h of incubation with PAR replacing it by 50 μL of DPBS. The λ_ex/em_ was of 490/530 nm. As a negative control, fluorescence of cells without DCFH-DA was measured in DPBS and then subtracted. Values were relativized to the positive control (untreated cells) and intracellular ROS increased was expressed as a fold change.

### 4.9. Flow Cytometry

MG63 and hBM-MSCs were seeded on 6-well plate at a density of 2 × 10^5^ per 2 mL of culture medium and incubated during 24 h. After that, cells were exposed during 2 h to 1 mL of PAR treated during 1, 2.5 and 5 min following the protocol in Figure 5. 24 h after treatment, cells were stained with Cell Death Apoptosis Kit with Annexin V Alexa Fluor™ 488 & Propidium Iodide (PI) (Invitrogen, cat.no 10257392, Carlsbad, CA, USA) following the manufacturer’s protocol. Cell counts were determined by flow cytometer BD LSR II and data analysis was performed with FlowJo Software. Each condition was done in triplicate.

### 4.10. Murine OS Organotypic Model

35 days old C57BL/6J mice were injected with 2 million MOS-J cells in 50 µL of DPBS by intramuscular paratibial injection. 17 days after injections, mice with 900–1200 mm^3^ tumor size were selected and sacrificed. Tumor sections of (2 × 2 × 0.2 mm) were obtained using LeicaVT 1200S vibratome under sterile conditions and sections were maintained in 2 mL of DMEM high glucose in 24 ultra-low attachment well-plates during 24 h. After that, tumor sections were exposed during 2 h to 1 mL Ringer’s saline that had been previously treated for 10, 15 or 20 min and then, PAR was diluted at ¼ in DMEM high glucose. Tumor sections were also exposed during 2 h to 500 µM H_2_O_2_ in Ringer’s saline with 10% of FBS and also were maintained with 100 µM Cisplatin (Santa Cruz, CA, USA) in DMEM high glucose. After 24 h of incubation, each treatment was repeated and tumor sections were kept in these for 48 more hours, being 72 h at the end. Sections from three different tumours were exposed to different conditions and 4 Sections were used per condition to estimate the mean. Tumor sections were incubated with resazurin solution (0.5 g/L Resazurin from Sigma in PBS) at 20% in DMEM high glucose during 3 h to measure metabolic activity. This was done before treatments and 24 and 72 h after first treatment. Fluorescence were measured with λex/em of 530/600 nm. As a negative control, fluorescence of resazurin without cells or tumor sections were measured and subtracted. Each value was relativized to control.

### 4.11. Histological Analysis

For histological analysis, tumor sections at 72 h after treatment were fixed in 4% formol overnight at 4 °C. After fixation, sections were dehydrated, included in paraffin, sectioned with microtome and deparaffinized before being rehydrated for histological analysis. Haematoxylin/eosin staining were performed to observe tissue structure and number of nuclei. For immunohistochemistry, sections were incubated in citrate buffer at 96 °C for 20 min and then blocked in PBS-Triton 0.05% with 1% BSA during 1 h at room temperature. After that, samples were washed with TBS-Tween 20 0.05% and then incubated with hydrogen peroxide 3% for 15 min to inhibit endogenous peroxidase. Sections were incubated with monoclonal rabbit anti-Ki-67 (Abcam, Cambrigde, UK) overnight at 4 °C. After that, samples were washed and incubated with secondary goat anti-rabbit biotinylated (Dako, E0432, Santa Clara, CA, USA) for one hour at room temperature. Then samples were incubated with streptavidin/peroxidase (Dako, P0397, Santa Clara, CA, USA) TBS-Tween 20 0.05% for one hour at room temperature. Diaminobenzidine (DAB) was used to visualize positive staining. Samples were countercolored with hematoxyline, washed and then mounted with Pertex. Images were taken by NanoZoomer (Hamamatsu Photonics). For positive nuclei area quantification and percentage of positive Ki-67 nuclei, 5 well-distributed high magnification pictures (X40) were taken for each condition in triplicate and analysed with ImageJ. For positive nuclei area, values were relativized to positive control. For percentage of positive Ki-67 nuclei, positive nuclei were quantified and relativized to total number of nuclei excluding the first 40 µm of tumor section (proliferating margins).

### 4.12. Statistical Analysis

All data are presented as means ± SD. Statistical analysis of the data was performed using ANOVA to compare conditions within the same experimental group and Student’s *t*-test to compare couple of conditions between them. *p*-values < 0.05 were considered statistically significant. For the case of viability and flow cytometry studies, *p*-values < 0.005 were considered statistically significant

## 5. Conclusions

Many studies reported that saline solutions activated by cold atmospheric plasma can offer a potential therapy against cancer, but there are very few works than demonstrate its effects in 3D and no studies in osteosarcoma models. The main aim of the present study was to demonstrate the cytotoxic effects of PAR in human and mouse osteosarcoma, employing 2D and organotypic cultures. Our data show that plasma-activated saline solutions could be used for the treatment of OS, by investigating the implication of RONS for the anti-cancer effect and cellular mechanisms involved in selective cell death. First, PAR induces cell death in adherent cell cultures, related to the increase in the intracellular ROS that triggers DNA damage and subsequent apoptosis, and not being related directly to H_2_O_2_ concentration. This kind of mechanism produced clearly higher lethal effects in osteosarcoma cells than in hBM-MSCs, that were significantly less affected. Second, PAR reduces OS tumor viability and proliferation, displaying a reduction of metabolic activity, number of nuclei and Ki-67 expressing cells. Although further investigations are needed, the results of the present work provide evidence of PAR as a promising tool for clinical use for OS treatment.

## Supplementary Materials

The following are available online at https://www.mdpi.com/2072-6694/12/1/227/s1, Figure S1: Time stability of H_2_O_2_ and NO_2_−produced; Figure S2: Effects on cell death by PAR treatment time in MG-63 cells and hBM-MSCs; Figure S3: Generation of NO_2_− and H_2_O_2_ by APPJ at the corresponding conditions for cell culture and organotypic protocols, Figure S4. Caspase-3 immunostaining in mouse tumor sections.
