# Supplementary material for: Cold Plasma-Treated Ringer’s Saline: A Weapon to Target Osteosarcoma
Source: Cancers (Basel). 2020 Jan 17;12(1):227. doi: 10.3390/cancers12010227 (PMC7017095; doi:10.3390/cancers12010227)
Supplement: Supplementary file 1 [file cancers-12-00227-s001.pdf]

Supplementary Materials:

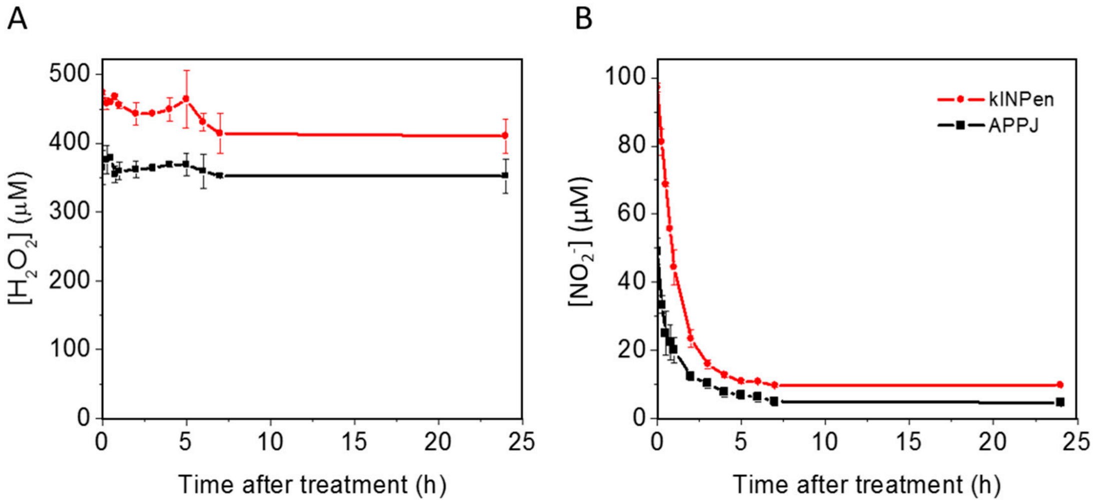

Figure S1. Time stability of (a) H_2O_2 and (b) NO_2^- produced in 2 mL Ringer's saline treated during 10 minutes with plasma jets APPJ (gas flow: 1 L/min) or kINPen (gas flow: 3 L/min) at a distance of 10 mm between the nozzle of the jet and the liquid surface.

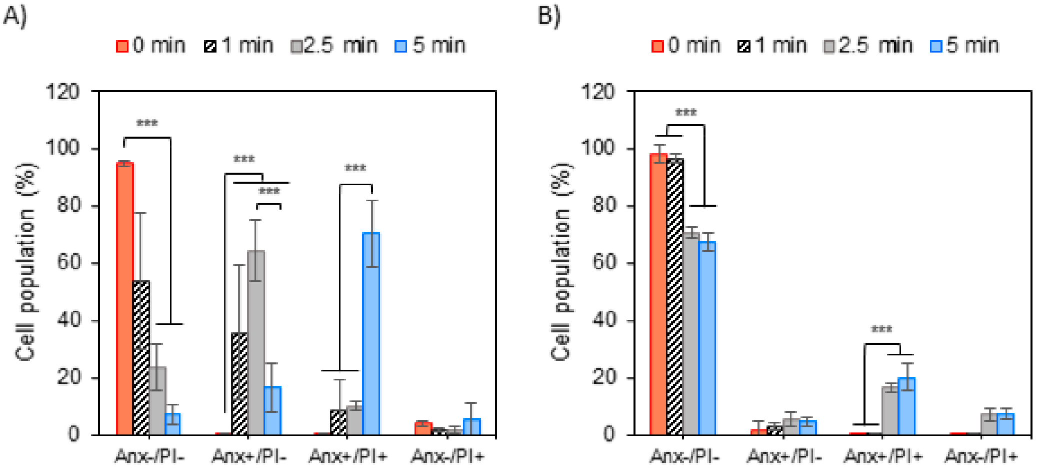

Figure S2. Effects on cell death by PAR treatment time in MG-63 cells and hBM-MSCs. MG-63 (A) and hBM-MSCs (B) in adherent culture were exposed during 2 hours to PAR (0-5 minuntes) and then PAR was replaced by fresh media. 24 hours after treatment, cells were stained with Annexin-V/PI and cell death was assessed by flow cytometry. Percentage of cells stained for Annexin-V and PI are represented. Asterisks represent statistically significance (n=3, ***$p < 0.005$; two-sided Student's t-test).

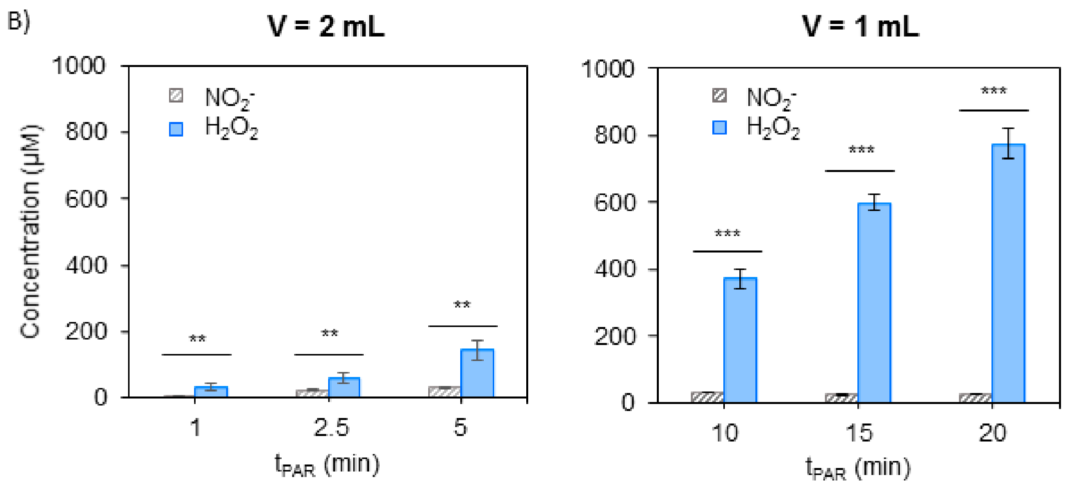

Figure S3. Generation of NO_2^- and H_2O_2 by APPJ at the corresponding conditions for cell culture and organotypic protocols. NO_2^- and H_2O_2 were determined by Griess reaction and Amplex Red test respectively (n=3, **$p < 0.01$; ***$p < 0.005$; two-sided Student's t-test).

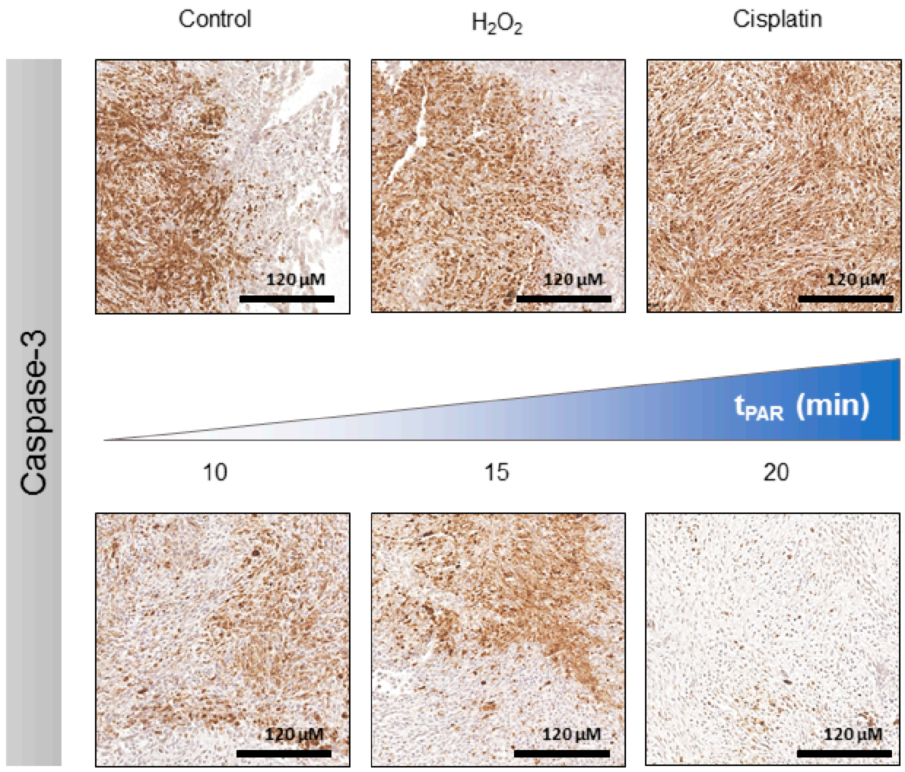

Figure S4. Caspase-3 immunostaining in mouse tumor sections. Mouse OS tumor sections in floating culture were treated with PAR at increasing treatment times and by 100 µM of Cisplatin and 500 µM of H_2O_2. Tumor sections were fixed 72 hours after treatment and processed for histological analysis. Samples were immunostained for Caspase-3. Representative images were taken at X20 (Scale bar = 120 µm).
